# Antiadhesive activity of poly-hydroxy butyrate biopolymer from a marine *Brevibacterium casei* MSI04 against shrimp pathogenic vibrios

**DOI:** 10.1186/s12934-014-0114-3

**Published:** 2014-08-13

**Authors:** George Seghal Kiran, Anuj Nishanth Lipton, Sethu Priyadharshini, Kumar Anitha, Lucia Elizabeth Cruz Suárez, Mariadhas Valan Arasu, Ki Choon Choi, Joseph Selvin, Naif Abdullah Al-Dhabi

**Affiliations:** Department of Food Science and Technology, Pondicherry University, Puducherry, 605 024 India; Department of Microbiology, Pondicherry University, Puducherry, 605 024 India; Consultor en Nutrición acuícola, Director Programa Maricultura, Facultad de Ciencias Biológicas Universidad Autónoma de Nuevo León, Cd. Universitaria, San Nicolas de los Garza, Nuevo León México; Department of Botany and Microbiology, Addiriyah Chair for Environmental Studies, College of Science, King Saud University, P. O. Box 2455, Riyadh, 11451 Saudi Arabia; Grassland and Forage Division, National Institute of Animal Science, RDA, Seonghwan-Eup, Cheonan-Si, Chungnam 330-801 Republic of Korea

**Keywords:** Vibrio pathogens, Anti-adhesive, vibriosis, Shrimp aquaculture

## Abstract

**Background:**

Vibrio pathogens are causative agents of mid-culture outbreaks, and early mortality syndrome and secondary aetiology of most dreadful viral outbreaks in shrimp aquaculture. Among the pathogenic vibrios group, *Vibrio alginolyticus* and *V. harveyi* are considered as the most significant ones in the grow-out ponds of giant black tiger shrimp *Penaeus monodon* in India. Use of antibiotics was banned in many countries due to the emergence of antibiotic-resistant strains and accumulation of residual antibiotics in harvested shrimp. There is an urgent need to consider the use of alternative antibiotics for the control of vibriosis in shrimp aquaculture. Biofilm formation is a pathogenic and/or establishment mechanism of Vibrio spp. This study aims to develop novel safe antibiofilm and/or antiadhesive process using PHB to contain vibrios outbreaks in shrimp aquaculture.

**Results:**

In this study a poly-hydroxy butyrate (PHB) polymer producing bacterium *Brevibacterium casei* MSI04 was isolated from a marine sponge *Dendrilla nigra* and production of PHB was optimized under submerged-fermentation (SmF) conditions. The effect of carbon, nitrogen and mineral sources on PHB production and enhanced production of PHB by response surface methods were demonstrated. The maximum PHB accumulation obtained was 6.74 g/L in the optimized media containing 25 g/L starch as carbon source, 96 h of incubation, 35°C and 3% NaCl. The highest antiadhesive activity upto 96% was recorded against *V. vulnificus*, and *V. fischeri*, followed by 92% against *V. parahaemolyticus* and *V. alginolyticus* and 88% inhibition was recorded against *V. harveyi*.

**Conclusion:**

In this study, a thermostable biopolymer was chemically characterized as PHB based on ^1^HNMR spectra, FT-IR and GC-MS spectra. The NMR spectra revealed that the polymer was an isocratic homopolymer and it also confirmed that the compound was PHB. The antiadhesive activity of PHB was determined in microtitre plate assay and an effective concentration (EC) of PHB (200 μl containing 0.6 mg PHB) was confirmed by confocal laser scanning microscopic analysis of vibrio biofilm on pre-treated glass and polystyrene surfaces. This is a first report on anti-adhesive activity of PHB against prominent vibrio pathogens in shrimp aquaculture.

**Electronic supplementary material:**

The online version of this article (doi:10.1186/s12934-014-0114-3) contains supplementary material, which is available to authorized users.

## Introduction

Gram negative *Vibrionacea* represent the most dreadful pathogenic bacteria causing disease in both grow-out ponds and hatcheries. In Asia, among the pathogenic vibrios group, 11 species were reported from the shrimp culture systems. Of these, *Vibrio alginolyticus* and *V. harveyi* are considered as the most significant ones in the grow-out ponds of giant black tiger shrimp *Penaeus monodon* in India [1]. Because of the development of resistance to the commonly used antibiotics, there is an urgent need to consider the use of alternative antibiotics for the control of vibriosis [1,2]. Use of antibiotics to control vibrios in shrimp aquaculture is not allowed in most of the countries and so it is necessary to develop an alternative pathogen control method for shrimp production [3]. Probiotics was considered as a sustainable method for shrimp disease control and health management. Alternate methods include novel antimicrobial patterns are being investigated to control vibrio pathogens in coastal aquaculture. Elastin-like biopolymer composed of a polyhistidine domain has been investigated as silver binding agent for antibacterial activity against *V. harveyi*, and concluded that artificial protein based antibacterial agent can be used in management of disease in coastal aquaculture [4]. Supplementation of formic acid in the shrimp feed as a control mechanism to contain vibrio outbreak in shrimp aquaculture system demonstrated for *V. alginolyticus, V. cholerae, V. harveyi, V. parahaemolyticus and V. vulnificus* [5].

Biofilms are sessile microbial community that is attached to a living or non-living surfaces and embedded in a self-produced matrix composed of extracellular polymeric substances [6]. These naturally existing biofilms are major threat to all life forms including fish/shrimp aquaculture. Bacteria occurring in biofilms are 10–1,000-fold more resistant to antibiotics than planktonic cells [7]. The highest antibiotic resistance was achieved by biofilm-forming bacteria due the creation of non-multiplying persistent subpopulations [8]. Biofilms formation is a succession of pathogenesis and survival/ escapes mechanism of pathogens against antibiotics and host immune system [9]. Thus, prevention or disruption of biofilms is inevitable to achieve effective treatment of planktonic cells. Literature reported that biofilm formation is a pathogenic and/or establishment mechanism of vibrio spp. [10].

Poly-β-hydroxybutyric acid (PHB) is a natural, biodegradable polymer accumulated in the form of intracellular granules by a large variety of bacteria [11]. These granules act as energy reserve materials when nutrients such as nitrogen and phosphorous source are available in limiting concentrations in the presence of excess carbon source [12,13]. The polymer can be made into films, fibres, and sheets, besides special applications in medical implants. More than 150 constituents of poly-hydroxy alkanoates (PHA) have been identified and characterized [14]. The PHB is one of the best characterized derivatives of PHAs. Since PHAs are of bacterial origin, and many bacteria and fungi can able to degrade these polymer to harmless products and the break down precursors are used for their own cell components and energy.

In this background, this study aims to develop novel safe antibiofilm and/or antiadhesive process using PHB to contain vibrios outbreaks in shrimp aquaculture. In this study, the effect of PHB as anti-adhesive agent against shrimp pathogens such as *Vibrio harveyi*, *V. alginolyticus*, *V. vulnificus*, *V. fischeri* and *V. parahaemolyticus* was evaluated. Rationale of this study was to test the effect of PHB on the prevention of vibrio adherence on different surfaces. This report describes the isolation and characterization of PHB polymer producing marine bacterium *Brevibacterium casei* MSI04. The effect of carbon, nitrogen and mineral sources on the PHB production and enhanced production of PHB by response surface methods were demonstrated. The purified PHB was characterized by FTIR, GC-MS and NMR spectra and thermal stability of the polymer was analysed by thermal gravimetric and differential scanning calorimetric analysis. This is a first report on anti-adhesive activity of PHB against prominent vibrio pathogens in shrimp aquaculture.

## Results

### Isolation, screening and identification of PHB producer MSI04

**Figure 1:**
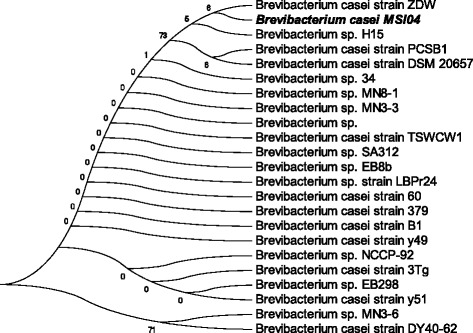
**Maximum parsimony phylogenetic tree of MSI04 and their closest NCBI (megaBLAST) relatives based on the 16S rRNA gene sequences.** Bootstrap values calculated from 1,000 resamplings using neighbor joining are shown at the respective nodes when the calculated values were 50% or greater.

In this study, 43 unique bacterial colonies were isolated from a marine sponge *Dendrilla nigra* but the stable isolates after subcultures were used in the screening. Among the 38 stable bacterial isolates from the marine sponge *D. nigra*, three strains (MSI04, MSI09, and MSI30) showed positive for PHB accumulation. The isolate MSI04 was noted as one of the effective producer of PHB based on the growth rate, the high intensity of fluorescence in viable Nile blue A staining, presence of lipophilic inclusions in Sudan black B staining, and dry weight of the extracted PHB (52%). The active producer was identified by cultural, morphological, biochemical characteristics and phylogenetic analysis of 16S rDNA sequence. The isolate was characterized as Gram-positive, aerobic, motile, transparent, mucoid and unelevated colony, hydrolyzing casein and gelatin and citrate positive. Taxonomic affiliation of the isolate MSI04 was retrieved from the *classifier* program of Ribosomal Database Project II (RDPII) (http://rdp.cme.msu.edu/) using 16S rDNA sequence. Representative of maximum homologous (97–99%) sequences of the isolate was obtained from *seqmatch* program of RDPII and were used reference strain in the construction of phylogenetic affiliation. The isolate MSI04 was clustered with *Brevibacterium casei* strain. The strain showed clustering with *Brevibacterium* sp. and *Brevibacterium casei* (Figure 1). Based on the biochemical, physiological and phylogenetic affiliation, the strain MSI04 was tentatively identified as *Brevibacterium casei.*

### Production of PHB under SmF

Maximum cell density was reached at 48 h of culture of *B. casei* MSI04 in SmF. The pH of the production medium was decreased from 7.5 to 5.6 during the growth of *B. casei* MSI04. The strain *B. casei* MSI04 can grow at a wide range of pH from 5 to 9 and maximum polymer accumulation (58% dry cell weight) was observed at pH 7.5 and 48 h of incubation at 30°C. At extremes of the temperature below 10°C and above 50°C the production was drastically declined. The strain *B. casei* MSI04 showed maximum synthesis of PHB in basal medium (nitrogen free) supplemented with starch as sole carbon source. The production was slightly decreased after the stationary phase. Based on the one-factor at a time experiments, the factors affecting the PHB production such as starch (g/L), incubation period (h), temperature (°C) and salinity (%) were selected for statistical optimization and interactive effect of these factors on PHB production was determined by response surface method (RSM). The CCD ANOVA suggests the selected factors were significant control factors for maximum production of PHB (Additional file 1: Tables S1 and S2). The maximum PHB accumulation obtained was 6.74 g/L with factors such as 25 g/L starch as carbon source, 96 h incubation, 35°C and 3% NaCl. The value of coefficient of determination (R^2^) was found to be 0.9887, which was validated in the production experiments showed the adequacy of the model (Additional file 1: Figure S1).

### Characterization of PHB polymer

**Figure 2:**
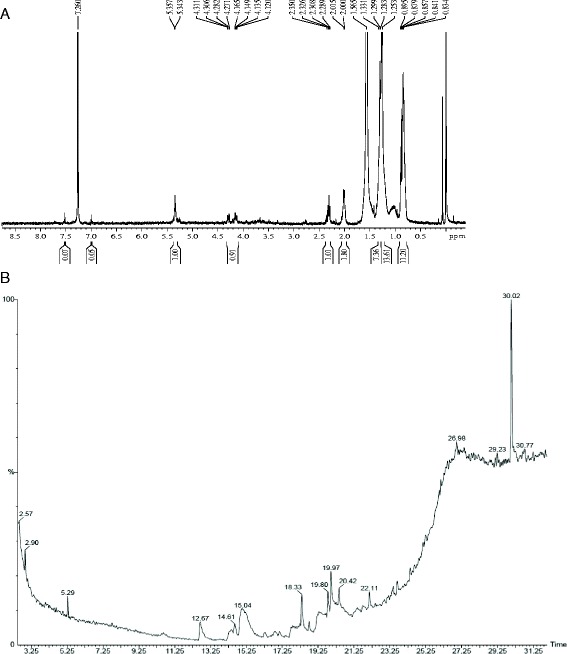
**Spectral characteristics of PHB. A**. ^1^HNMR spectra of PHB produced from *Brevibacterium* sp. **B**. GC-MS spectra of polymer produced by MSI04 resembling methyl 3-hydroxy butyrate with the retention time of 2.57 min.

The highest UV scanning intensity was recorded at the PHB wavelength (235 nm) with maximum absorbance of 1.65. SEM analysis evidenced accumulation of PHB granules in the medium and PHB polymer extracted using sodium hypochlorite followed by chloroform and acid treatment (Additional file 1: Figure S1). The IR spectroscopic analysis gave further insights into the chemical structure of the polymer and reflects the monomeric units. The FT-IR analysis showed peaks around 3446 – 3455 cm^−1^ indicating -OH stretching, around 2922 – 2850 cm^−1^ corresponds to methylene attached with oxygen aliphatic –CH. The peak obtained around 1740–1760 cm^−1^ indicates the presence of C = O ester linkage. The peaks around 1038 cm^−1^ may be because of HC-O vibrations and peaks obtained around 1460–1419 cm^−1^ may be of α – CH_2_ bending vibration. The polymer extracted from *B. casei* MSI04 was analyzed by GC-MS to determine the constituent acids present in the polymer. Gas chromatography analysis revealed the major peak, resembling to methyl 3-hydroxybutyrate with the retention time of 2.57 min, which confirms the synthesis of PHB in *B. casei* MSI04 (Figure 2). The ^1^HNMR spectra of PHB from *B. casei* MSI04 was extrapolated with major peaks at 1.23, 2.5 ppm and 5.2 ppm, which was due to the resonance absorption of methyl (CH_3_), methylene (CH_2_) and methane (CH) groups, respectively, in 3-hydroxybutyrate (3-HB). The NMR spectra revealed that the polymer as an isocratic homopolymer and it also confirmed that the compound was PHB (Figure 2).

### Differential scanning calorimetric (DSC) and X-Ray Diffraction (XRD) patterns

**Figure 3:**
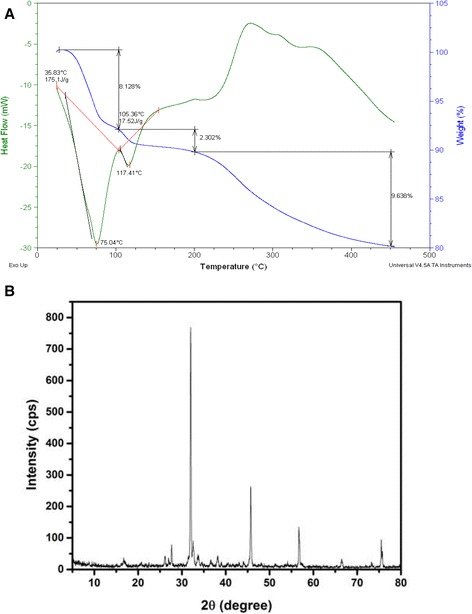
**Thermal stability and crystalline nature of PHB. A**. TG and DSC thermogram of PHB obtained from *Brevibacterium* MSI04. **B**. XRD patterns of PHB.

Thermal stability of PHB is an important characteristic for its commercial utilization. Degradation of PHB took place by three well differentiated steps as observed in TG analysis (Figure 3A). Eight percent (8.128%) of weight loss was recorded from 35 to 106°C due to loss of moisture molecules followed by second phase degradation at 200°C with a slight weight loss of 2.302% was observed and maximum degradation at 450°C with a weight loss of 9.638%. The DSC thermogram showed exothermic peak of PHB with crystallization temperature (*T*_c_) of 117.41°C accompanied with 175.1 J latent energy. The melting peaks were found at 225°C and 350°C respectively.

The XRD spectra of the sample showed the crystallization pattern of PHB, which was consistent with the DSC thermogram. The profile exhibits well-defined peaks (2θ) at 17.1°, 27.6°, 32.7°, 46.5°, 58.2° which corresponds to the reflections of the orthorhombic crystalline lattice (Figure 3B). The XRD analysis was an evidence to show crystallinity of PHB decrease with the increases in the HHx content. The diffraction profile of PHB sample was almost similar to PHB homopolymer.

### Analysis of antiadhesive activity of PHB

**Figure 4:**
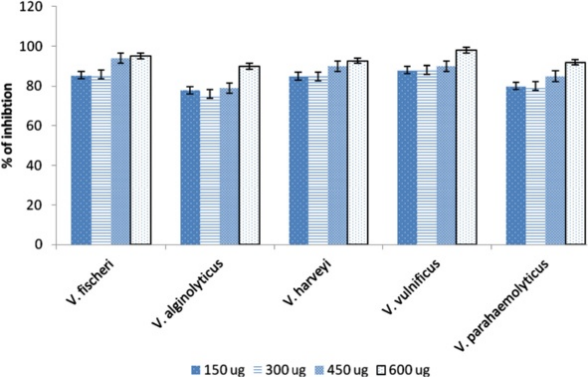
**Percentage of antiadhesive activity of PHB on various** ***Vibrio*** **spp.** The inhibition of PHB was found at the concentration 200 μl against *V. fischeri, V. alginolyticus, V. harveyi, V. vulnificus,* and *V. parahaemolyticus.*

The effect of the PHB on biofilm formation of vibrios was studied using microtitre plate assay, light microscopic and phase contrast microscopic observations. The control biofilms showed a higher surface coverage, whereas, in the PHB coated wells showed significant decrease in the biofilm formation of *Vibrio* spp. The preliminary phase contrast microscope analysis revealed reduction of biofilm formation on the PHB coated glass slide over the control and standard PHB (Additional file 1: Figure S3). Therefore, the antiadhesive activity of *Brevibacterium* PHB was demonstrated with plate assays and CLSM analysis. Microtitre plate assay showed significant reduction in biofilm formation in the PHB coated glass and polystyrene plates. The assay evidenced that 600 μg of PHB was effectively prevented the adhesion of vibrios when compared to other concentrations (150 μg, 300 μg, and 450 μg) tested (Figure 4). The highest antiadhesive activity upto 96% was recorded against *V. vulnificus*, and *V. fischeri*, followed by 92% against *V. parahaemolyticus* and *V. alginolyticus* and 88% inhibition was recorded against *V. harveyi*. The highest reduction of adhesion (88-96%) was observed with 600 μg of PHB. It was also observed that the dislodging effect of PHB on preformed biofilms was lower by (50%) when compared with the preventive effect of pre-coated PHB on the surfaces. Results of microtitre plate assay suggest that when the surface was covered by PHB the adhesion was inhibited effectively.

### Confocal laser scanning microscopy

**Figure 5:**
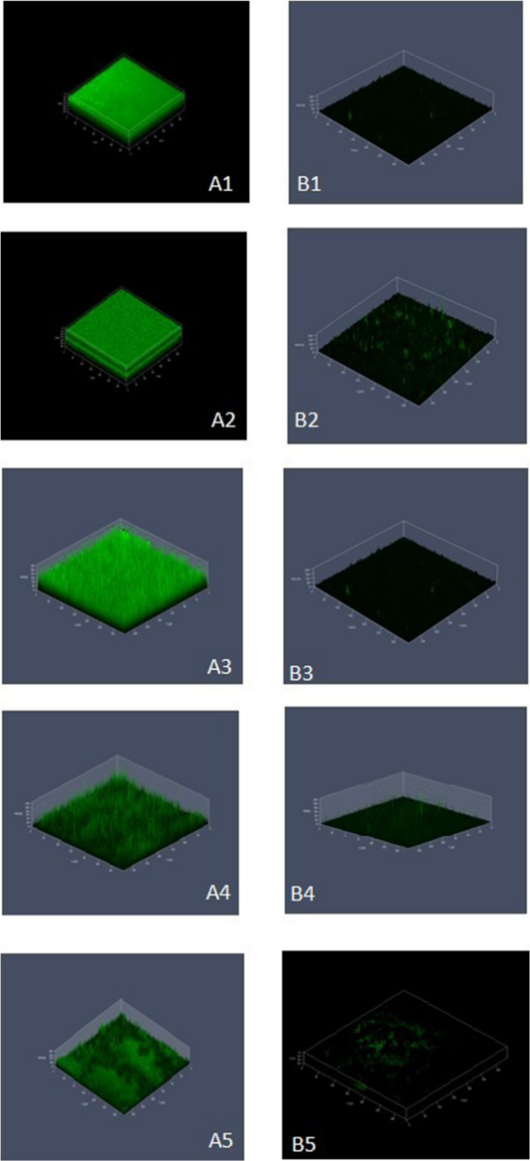
**Confocal laser scanning micrographs of** ***Vibrio*** **.** biofilms formed on glass surface. A1-A5. Biofilm formed on glass surface (control). B1-B5. Effect of PHB precoated on the glass surfaces. The PHB coated glass surfaces showed least or no formation of biofilm evidenced the effect of PHB on the control of *Vibrio* biofilm. A1 & B1 are control and treated biofilm of *Vibrio harveyi,* A2 & B2 are *Vibrio parahaemolyticus,* A3 & B3 are *Vibrio fischeri,* A4 & B4 are *Vibrio alginolyticus* and A5 & B5 are *Vibrio vulnificus.*

**Figure 6:**
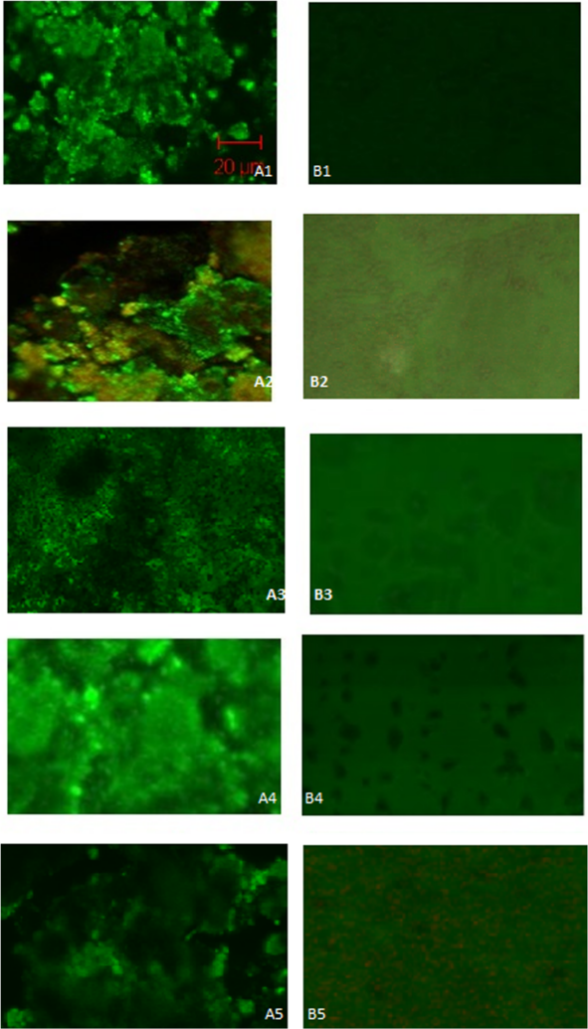
**Confocal laser scanning micrographs of** ***Vibrio*** **biofilms formed on polystyrene surface.** A1-A5. Biofilm formed on polystyrene surface (control). B1-B5. Effect of PHB precoated on the polystyrene surfaces. A1 & B1 are control and treated biofilm of *Vibrio harveyi*, A2 & B2 are *Vibrio parahaemolyticus*, A3 & B3 are *Vibrio fischeri*, A4 & B4 are *Vibrio alginolyticus* and A5 & B5 are *Vibrio vulnificus*.

The antiadhesive effect of PHB on pathogenic *Vibrio* was confirmed by CLSM analysis of PHB coated glass slides and polystyrene plates. Based on the CLSM analysis, it was confirmed that the PHB effectively prevented adhesion of *Vibrio* on glass and polystyrene surfaces coated with 600 μg PHB. The uncoated glass/polystyrene slides served as control which showed fully covered biofilm (Figure 5 and 6). The PHB coated glass surfaces showed least or no formation of biofilm evidenced the effect of PHB on the control of *Vibrio* biofilm (Figure 5). Similar antiadhesive effect of PHB was observed on pre-coated polystyrene surface (Figure 6). The PHB coated glass and polystyrene plates showed 80-95% antiadhesive effect on all tested *Vibrio* spp*.* The highest antiadhesive activity was recorded against *V. vulnificus* and *V. fischeri* followed by *V. parahaemolyticus, V. alginolyticus* and *V. harveyi* (Figure 5 and 6)*.* The antiadhesive activity of PHB was effective on both glass and polystyrene surfaces.

## Discussion

Vibrios are ubiquitous in environments mainly aquatic ecosystems. The biofilm formation was a well-documented mechanism of vibrio pathogens in general [10]. The biofilm forming capacity of *V. cholerae* was well established in *in vitro* and *in situ* conditions [10 and references therein]. But the biofilm formation capacity and its effect on pathogenesis in shrimp was not comprehensively demonstrated for vibrio pathogens of shrimp aquaculture. Several studies have suggested that biofilm formation is a survival mechanism, pathogenesis, and stress tolerance of *Vibrio* spp. [10]. To date, only few studies have been carried out on the biofilm inhibition of *Vibrio* spp. [15,16].

In this study a potential PHB producer *B. casei* MSI04 was isolated from a marine sponge *D. nigra*. Marine sponges are sedentary animals harbour bacteria up to 40% of their biomass [17]. Marine sponge associated bacteria can be a potential source of PHB producers since the nutrient limitation in the sponge mesohyl (niche) perhaps facilitate the synthesis of PHB granules [18]. In general marine ecosystems are unique habitat of microbes which are exposed to a wide variety of environmental conditions including extremes in temperature, salinity, nutrient limitation and pressure. Survival under such stress conditions must have necessitated to evolve adaptation and development of unique cellular biochemistry and metabolism by these microbes which may facilitate the synthesis of biopolymers. The extra-cellular product of marine actinomycetes was effectively inhibited biofilm formation of vibrio [15]. Sponge associated marine bacteria have already been exploited for the production of biopolymers, antibiofilm biosurfactants, and exopolysaccharides [16,18].

The scope of the present study include production, optimization, characterization, and evaluation of PHB produced by *B. casei* MSI04 and development of safe alternate to antibiotics for the control of vibrio pathogens in shrimp aquaculture. We have isolated a new effective PHB producer *B. casei* MSI04 from a marine sponge *D. nigra*. The PHB accumulation was started at the log phase and the maximum synthesis of PHB was reached at stationary phase of growth. The PHB accumulation was slightly decreased after the stationary phase due to the intracellular utilization of the PHB as energy and carbon reserves. The advantage of RSM was the study of interaction between the coded variables such as starch, incubation period, temperature and salinity which could not be achieved in conventional one-factor-at-a-time method [19]. The production PHB by *B. casei* MSI04 was optimum at pH 7.5. But higher pH of 8.0 and 9.0 respectively were reported as optimum for PHB production [20,21]. The isolate MSI04 was halo-tolerant, it can grow well up to 12% NaCl and the production maximum was attained with 3% NaCl. The maximum production of PHB in SmF was 6.74 g/L with optimized factors such as starch, incubation period, temperature and salinity. The crystallinity of the polymer was interfered with side chains of the polymer, the existence of different units and their irregular array. The results were similar to the results of medium chain length poly hydroxy alkanoates [22]. The sharper peak indicates the crystalline nature of PHB and the peak intensities of 760 cps were lesser than the standard PHB recorded by De Rooy et al. [22]. Highly crystalline polymers were usually stiff and brittle resulting in very poor mechanical properties with low extension at break [23] but they were very low resistance to thermal degradation [24]. According to Sato et al. [25], the P(HB-co-HHx) copolymer has a propyl side chain which imparts flexibility and compatible with other polymers. The chemical characteristics of PHB were matching with our previous report [18].

The polymer obtained in this study was heat stable and insoluble in water. The adhesion of pathogenic vibrio to polystyrene surfaces and glass was inhibited by PHB at concentration of 0.6 mg/200 μl. We found the PHB act as a strong anti-adhesive agent (80-95%) against all tested vibrio pathogens. This study evidenced that the pathogenic vibrio in shrimp aquaculture can be effectively controlled by PHB. Therefore, PHB can be applied to the shrimps as feed additive/coat which can reduce the colonization capacity of vibrio on the farmed shrimp as well as in the farm environment. Vibrios are important bacterial pathogens for animals reared in aquaculture. *V. alginolyticus, V. salmonicida, V. parahaemolyticus* and *V. vulnificus* are among the main bacterial pathogens of several fish/shrimp spp. [26]. Lee et al. [27] evaluated cell adhesion of various aliphatic polyesters and they found that (RS)-PHB have low cell adhesion because of the surface accumulation of methyl groups. Defoirdt et al. [28] reported that short-chain fatty acids like PHB showed a strong growth-inhibitory against pathogenic *V. campbellii*. Recently PHB was proven to be an immunostimulant in fish [29]. The synergistic effect of PHB-degrading probiotic and PHB as a synbioticum elicits a strong protection against luminescent vibriosis [30]. PHB increases growth rate and act as symbiotic of intestinal microglora of fish [31]. Therefore, the use of PHB in shrimp aquaculture could be biocompatible and elicit synergistic effect as antiadhesive as well as possibly as immunostimulant in treated shrimps.

## Conclusion

Control of vibrio infection by disrupting/preventing their biofilm formation would reduce the biomass establishment, a prerequisite step for virulence expression. Thus prevention of biofilm formation instead of inhibiting growth, could serve as an alternative to conventional procedures [32]. The significant potential of PHB in shrimp aquaculture remain a promising field of research require comprehensive investigation. In this study, we achieved maximum production of PHB in SmF was 6.74 g/L with starch as sole carbon source. Therefore, an economic production process can be attained for promoting PHB supplement in shrimp feed as a package of practice. In conclusion PHB was a potential antiadhesive agent which can be developed as a safe economic alternate to replace conventional antibiotics in shrimp aquaculture.

## Materials and methods

### Isolation and screening of PHA producer

The marine bacteria were isolated from a marine sponge *D. nigra* as described by Selvin et al. [17]. Briefly, 1 cm^3^ of surface-sterilized sponge tissue was excised and the excised portion was thoroughly washed three times in sterile aged seawater to remove any bacteria within current canals. The tissue was homogenized with phosphate buffered saline using a tissue homogenizer. The resultant homogenate was serially diluted with sterilized aged seawater and preincubated at 40°C for 1 h for the activation of dormant cells. The aliquot was placed on various isolation media including marine sponge agar [33]. The inoculated plates were incubated at 30°C for 7 days in dark. The morphologically distinct colonies were reisolated and maintained on Zobell marine agar (HiMedia) at 4°C. The isolates were designated as marine sponge isolates (MSI). The isolated bacteria were screened for PHA production using viable colony staining method. Briefly, Zobell marine agar supplemented with solution of 0.25 mg Nile blue A (w/v in dimethyl sulfoxide) to give final volume of 0.5 μg dye ml^−1^ medium. The colonies were directly examined for fluorescence under UV light to detect the accumulation of PHA. The efficient PHA producer was identified by Sudan black B staining method.

### Characterization of PHA producer

The morphological and biochemical characteristics of the pure isolate (MSI04) containing lipophilic inclusions were identified according to Bergeys’s manual of determinative bacteriology [34]. For molecular characterization, the genomic DNA was extracted from 2 ml of pure culture using the method of Enkicknap et al. [35] with suitable modifications. From the genomic DNA, nearly full-length 16S rRNA sequences were amplified using the primers 8 F (5′- AGA GTT TGA TCC TGG CTC AG-3′) and 1492R (5′- GGT TAC CTT GTT ACG ACT T-3′). The amplicon was cloned using TOPO TA Cloning kit according to manufactures instructions (Invitrogen) for sequencing. The 16S rDNA gene sequence obtained from the isolate MSI04 was compared with other bacterial sequences using NCBI megaBLAST (http://blast.ncbi.nlm.nih.gov/Blast.cgi) for their pair wise identities. Multiple alignments of these sequences were carried out by ClustalW 1.83 version of EBI (http://www.ebi.ac.uk/Tools/msa/clustalw2/) with 0.5 transition weight. Phylogenetic tree was constructed in MEGA 6.0 version (www.megasoftware.net) using maximum parsimony algorithm. Nucleotide composition of each aligned sequence was predicted by BioEdit software package.

### PHB production under submerged fermentation

The isolate MSI04 was allowed to grow in 50 ml of Luria Bertani (LB) broth (1% casein hydrolysate; 0.5% yeast extract; 1% NaCl; pH 7.5) in a 250 ml Erlenmeyer flask, at 28°C for 24 h, aerobically. The seed culture (5% v/v), having a cell density of 10^6^ cells/ml, was inoculated into 500 ml of modified production medium containing (NH_4_)_2_SO_4_ 1 g/L, K_2_HPO_4_ 5.72 g/L, KH_2_PO_4_ 3.7 g/L, citrate 5.87 g/L, phenyl acetic acid 2.72 g/L MgSO_4_.7H_2_O 0.5 g/L, Ca(NO_3_)_2_ 0.01 g/L with 5 or 10% (w/v) glucose. Batch fermentation was performed in flasks at 28°C with shaking at 200 rpm. All experiments were carried out in triplicates.

### Extraction and quantification of PHB

Extraction of PHB was carried out by centrifugation of the broth at 10,621 × g for 20 min (Eppendorf). The pellet obtained was lyophilized and digested with 30% sodium hypochlorite solution at 37°C for 30 min. The sample was then centrifuged at 8,000 × g for 30 min and washed sequentially with distilled water (5 ml), acetone (5 ml) and methanol (5 ml). Finally the residue was extracted with boiling chloroform and filtered through Whatman No. 1 filter paper. The chloroform extract was evaporated to dryness and the sample was further treated with concentrated H_2_SO_4_ and incubated at 100°C for 30 min. Absorbance of the resultant solution was measured at 235 nm using a UV-visible light spectrophotometer (PG Instruments) with crotonic acid as the standard. The PHB quantification was carried out using the method of Law and Slepecky [36].

### Optimization of PHB production

Optimization of PHB production was carried out by search one parameter at a time technique. Subsequently appropriate experimental models were developed in order to study the interactions between the factors. Factors such as carbon and nitrogen sources, pH, temperature, incubation time and salt concentration affecting the PHB production were determined. The optimization process was completed with effective concentration of chosen carbon and nitrogen source to maximize the production under submerged fermentation (SmF) conditions. Factors affecting the production including carbon sources (sucrose, glucose, fructose and starch), nitrogen sources (NH_4_NO_3_, yeast extract, beef extract and glycine), temperature (4 to 60°C), salinity (1 to 15% w/v), and pH (5–9) were included in the optimization. Uninoculated flasks and flasks without substrates(s) were included as controls.

### Statistical optimization of PHB production

The effective media components and environmental factors on the production of PHB were selected based on single factor experiments. The critical control factors were optimized statistically with central composite design (CCD). The objective of this experiment was to develop an empirical model of the process and to obtain a more precise estimate of the optimum operating conditions for the factors involved. Based on the first-order model equation obtained by the fractional factorial design, a series of trials were performed in the direction of the steepest ascent. In order to fit empiric second-order polynomial model, the CCD with 6 coded levels was performed. The quadratic model for predicting the optimal point was expressed according to the following equation:

$$ Y={b}_0+{\displaystyle \sum {b}_i{x}_i+{\displaystyle \sum {b}_{i i}{\displaystyle {x}_i^2}}+{\displaystyle {b}_{i j}}{\displaystyle {x}_j}{\displaystyle {x}_j}} $$

Where y is the response variable, b is the regression coefficients, and x is the coded level of the independent variable. A central composite design coupled with a full quadratic polynomial model is a very powerful combination that efficiently provides an adequate representation of most continuous response surfaces without expending many resources. Moreover, a better understanding of the process and the variables under study helps achieve a more realistic model. Thus, a 2^4^ full factorial central composite design for four test variables, each at three levels with six replicates at the centre points was employed to fit a quadratic model, indicated that 30 experiments were required for the procedure. The factors chosen for the experimental design were as follows: starch (g L^−1^), NaCl (gL^−1^), temperature, and incubation time. The optimal values of the experimental conditions were obtained by solving the regression equation and the response-surface contour plots. All statistical analysis was carried out by software package of Design-Expert® v. 8.0.7.1 (Stat Ease Corporation, USA).

### Detection and characterization of PHB

PHB granules were detected using SEM microscopy, and the polymer obtained after extraction was air dried, coated with platinum vapour and observed under a scanning laser electron microscope (Hitachi, Model S-3400 N). The images were acquired at an accelerating voltage of 10–15 kV and magnification of 7,000×. It can also be analysed by monitoring UV spectra of extracted PHB by scanning between 220 and 300 nm, and compared with standard PHB which has highest absorbance at 235 nm.

The purified PHB was thoroughly mixed with KBr and dried. The dried sample was subjected to IR spectrum using Fourier Transform Infrared Spectrophotometer (Thermo Nicolet Model:6700) with spectral range of 4000–400 cm^−1^. The polymer was investigated by using Gas chromatography (GC) (Perkin Elmer Autosystem XL GC-model Clarus-680, USA).The lyophilized cell mass was subjected to methanolysis and the resulting methyl esters of PHB were assayed. Briefly, 1 μl of sample in chloroform were injected with helium (1 ml min^−1^) as the carrier gas. The injector temperature was maintained at 250°C and the column temperature was increased from 40 to 240°C at 20°C/min and held at the final temperature for 10 min. The peaks of the gas chromatography were subjected to mass-spectral analysis and the spectra were analyzed by NIST MS search (version 2.0). ^1^H NMR spectra was acquired by dissolving the PHB in deuteron chloroform (CDCl_3_) at a concentration of 20 mg/ml and analyzed on a Bruker Model: Avance-II at 22°C .

### Thermal gravimetric (TG), differential scanning calorimetric (DSC) and X-Ray Diffraction (XRD) analyses

Thermal stability and weight loss of PHB was performed and determined using TA instruments, Q600 SDT and DSC. Approximately 16–20 mg of sample was placed in standard 70 μl aluminium pans. The analysis was carried out over the temperature range from 0°C to 500°C at a rise in temperature of 10°C/min. The flow rate of the gas was 50 ml/min. The weight loss is recorded as a function of temperature.

The degree of crystallinity was estimated using X - ray diffraction (XRD) patterns obtained using a Rigaku (30 kv/25 mA) Geigerflex D/Mac, C series diffract meter (Tokyo, Japan) with Cu-kα radiation (λ = 1.5406A°) at room temperature. The scanning range was 10-0° at a rate of 0.02°/second. The sample was first hot pressed at 100° for 2 min to erase the residual thermal history, and subsequently quenched to room temperature, followed by maintaining for 24 h to allow complete crystallization.

### Antiadhesive activity of PHB on vibrio pathogens

The isolates such as luminescent *V. harveyi*, *V. alginolyticus*, *V. vulnificus*, *V. fischeri*, and *V. parahaemolyticus* were experimentally proven as potential shrimp pathogens and were used in the present study. Briefly, during September 1999, an extensive shrimp farm located in southeast coast of India was affected by shell disease in cultured shrimp *Penaeaus monodon*. However, no mortality was observed until the sampling day (45 d of culture and 2 d after the report of disease incidence). The details of isolation and characterization of vibrio pathogens was reported in our previous work [26].

Preliminary assay was set with standard PHB (Sigma) and *Brevibacterium* PHB coated glass slide. The antiadhesive activity of the *Brevibacterium* PHB on vibrio pathogens was tested according to the procedure described by Heinemann et al. [37] with suitable modifications. Briefly, the wells of a sterile 96-well flat-bottom plate were coated with 50, 100, 150, and 200 μl of PHB. The concentration of PHB in 100 μl was 0.3 mg, accordingly the effective concentration was calculated. The PHB coated plates were allowed to dry at 40°C in an incubator and subsequently washed twice with PBS. Negative and growth control wells did not contain PHB. All wells (except for negative controls) were inoculated with overnight cultures of *Vibrio* spp. Inoculum was prepared using overnight broth culture. The cultures were centrifuged, washed twice with phosphate buffered saline (PBS, pH 7.4) and re-suspended in PBS to obtain OD_600_ = 1.0. A 100 μl aliquot of a washed vibrio suspension was added to the wells and incubated for 2 h at 37°C in a rotary shaker and the non-adherent cells were removed by repeated washes with PBS. The adhered vibrio was fixed with 100 μl of methanol (Sigma) per well and the wells were air-dried. Then the plates were stained with 100 μl of 2% crystal violet for 7 min. Excess stain was washed with running tap water and air-dried. The adherent vibrio cells were resolubilized in 100 μl of 33% (v/v) glacial acetic acid in each well. The OD was determined at 595 nm in a microplate reader (Labnics). The percent inhibition of vibrio adherence was calculated as per the method of Gudiña et al. [38].

$$ \%\mathrm{of}\;\mathrm{vibrio}\;\mathrm{inhibition}=\left[1-\left( T/ C\right)\right]\times 100 $$

Where, T stands OD value of PHB treated and C stands for OD value of control well.

The plate was observed under a phase-contrast microscope with ×40 magnification (Optica) to evaluate the biofilm formation. The microbial inhibition was calculated as area of growth (biofilm) on the slide. All assays were carried out in replicates.

### Confocal laser scanning microscopy (CLSM)

Based on the microtitre plate assay, the effective concentration (EC) of PHB was confirmed by CLSM analysis of biofilm formation on pre-treated glass and polystyrene surfaces. The EC of PHB 200 μl was coated on the glass cover slips and polystyrene surfaces and exposed to vibrio culture. The preformed biofilm on both surfaces were stained with 0.1% acridine orange and observed under CLSM (LSM 710, Carl Zeiss, Germany). The 488-nmAr laser and 500–640-nm band pass emission filter were used to excite and detect the stained cells. Multiple (20) images were scanned and processed slides using Zen 2009 image software.

## Additional file

Additional file 1: Table S1. Central Composite Design (CCD) design with experimental yield of PHB. **Table S2.** ANOVA for response surface quadratic model of PHB yield. **Figure S1.** Maximum parsimony phylogenetic tree of MSI04 and their closest NCBI (megaBLAST) relatives based on the 16S rRNA gene sequences. Bootstrap values calculated from 1,000 resamplings using neighbor joining are shown at the respective nodes when the calculated values were 50% or greater. **Figure S2.** SEM images of PHB polymer A. Extracted PHB and B. PHB granules accumulated in the medium. **Figure S3.** The phase contrast microscope images of biofilm formed on glass slides. A. Control biofilm of *V. parahaemolyticus*, B. *Brevibacterium* PHB coated glass slide, C. Standard PHB coated glass slide.
