# Correction to: Antiadhesive activity of poly-hydroxy butyrate biopolymer from a marine *Brevibacterium casei* MSI04 against shrimp pathogenic vibrios

**DOI:** 10.1186/s12934-020-01484-0

**Published:** 2021-01-21

**Authors:** George Seghal Kiran, Anuj Nishanth Lipton, Sethu Priyadharshini, Kumar Anitha, Lucia Elizabeth Cruz Suárez, Mariadhas Valan Arasu, Ki Choon Choi, Joseph Selvin, Naif Abdullah Al-Dhabi

**Affiliations:** 1 Department of Food Science and Technology, Pondicherry University, Puducherry, 605 024 India; 2 Department of Microbiology, Pondicherry University, Puducherry, 605 024 India; 3 Consultor en Nutrición Acuícola, Director Programa Maricultura, Facultad de Ciencias Biológicas, Universidad Autónoma de Nuevo León, Cd. Universitaria, San Nicolas de los Garza, Nuevo León México; 4 Department of Botany and Microbiology, Addiriyah Chair for Environmental Studies, College of Science, King Saud University, Riyadh, 11451 Saudi Arabia; 5 Grassland and Forage Division, National Institute of Animal Science, RDA, Seonghwan-Eup, Cheonan-Si, 330-801 Chungnam Republic of Korea

## Correction to: Microb Cell Fact (2014) 13:114 https://doi.org/10.1186/s12934-014–0114-3

The authors regret the use of incorrect panels in Fig. 5 (B1) and Fig. 6 (A2, A4, and A5) of their publication [1]. These errors affect neither the research data presented nor the article conclusion.

The corrected Figs. 5 and 6 are provided in this correction.

**Fig. 5:**
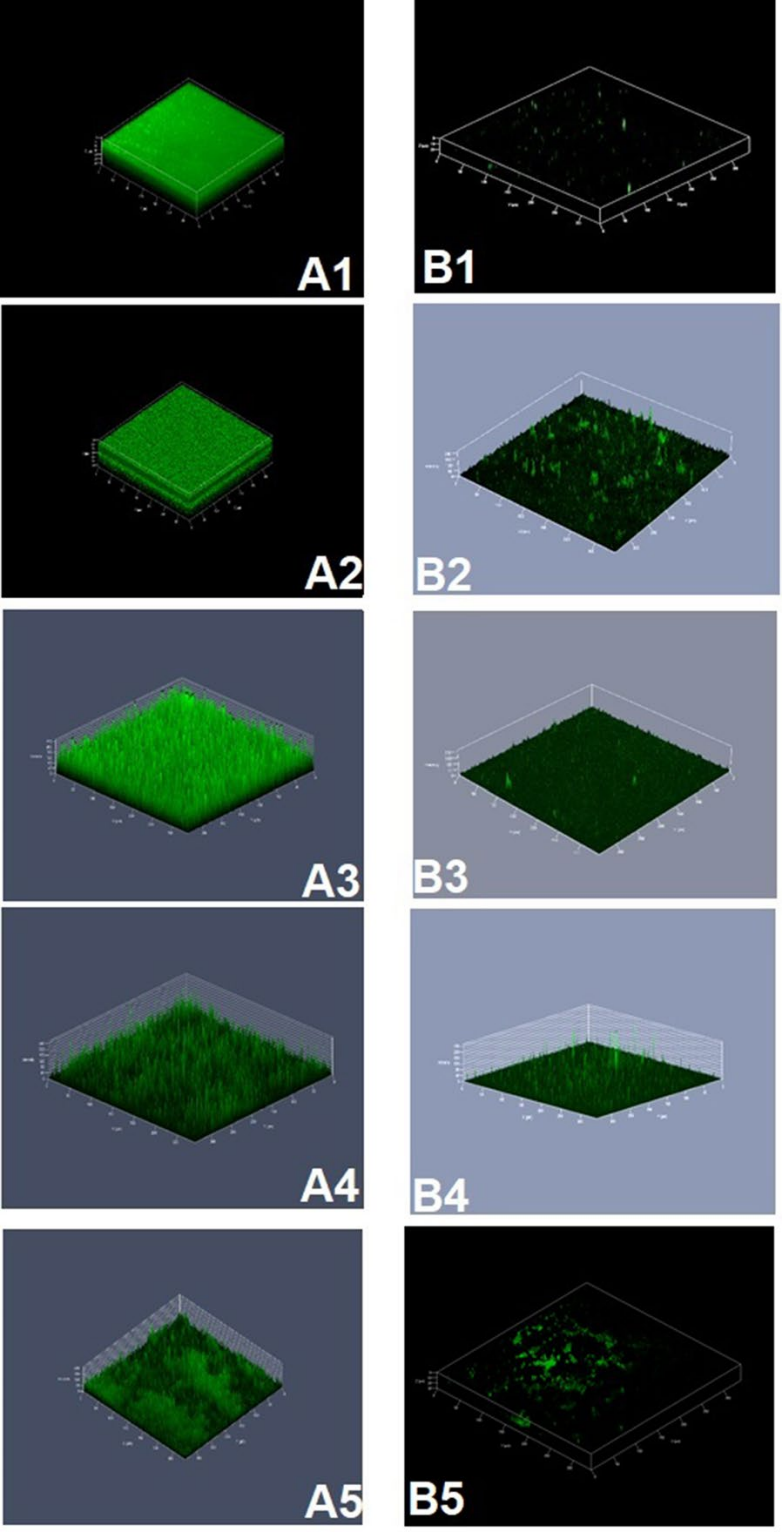
Confocal laser scanning micrographs of *Vibrio* biofilms formed on glass surface. **A1**−**A5**. Biofilm formed on glass surface (control). **B1**−**B5**. Effect of PHB precoated on the glass surfaces. The PHB coated glass surfaces showed least or no formation of biofilm evidenced the effect of PHB on the control of *Vibrio* biofilm. **A1** & **B1** are control and treated biofilm of *Vibrio harveyi,* **A2** & **B2** are *Vibrio parahaemolyticus,* **A3** & **B3** are *Vibrio fischeri,* **A4** & **B4** are *Vibrio alginolyticus* and **A5** & **B5** are *Vibrio vulnificus*

**Fig. 6:**
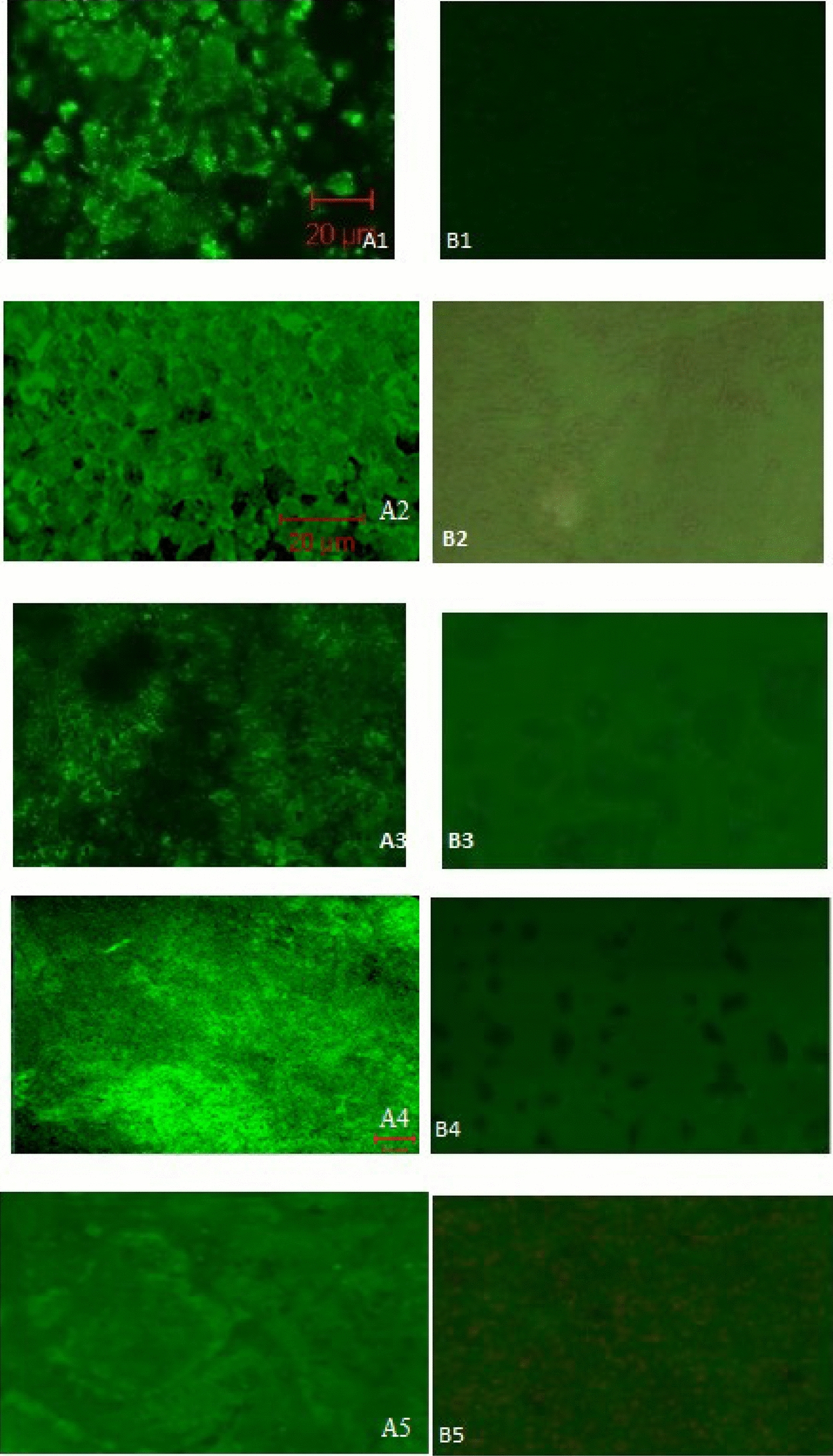
Confocal laser scanning micrographs of *Vibrio* biofilms formed on polystyrene surface. **A1**−**A5**. Biofilm formed on polystyrene surface (control). **B1**−**B5**. Effect of PHB precoated on the polytyrene surfaces. **A1** & **B1** are control and treated biofilm of *Vibrio harveyi,* **A2** & **B2** are *Vibrio parahaemolyticus,* **A3** & **B3** are *Vibrio fischeri,* **A4** & **B4** are *Vibrio alginolyticus* and **A5** & **B5** are *Vibrio vulnificus*
