# Supplementary material for: Antiadhesive activity of poly-hydroxy butyrate biopolymer from a marine Brevibacterium casei MSI04 against shrimp pathogenic vibrios
Source: Microb Cell Fact. 2014 Aug 13;13:114. doi: 10.1186/s12934-014-0114-3 (PMC4149260; doi:10.1186/s12934-014-0114-3)
Supplement: Additional file 1: Table S1. — Central Composite Design (CCD) design with experimental yield of PHB. Table S2. ANOVA for response surface quadratic model of PHB yield. Figure S1. Maximum parsimony phylogenetic tree of MSI04 and their closest NCBI (megaBLAST) relatives based on the 16S rRNA gene sequences. Bootstrap values calculated from 1,000 resamplings using neighbor joining are shown at the respective nodes when the calculated values were 50% or greater. Figure S2. SEM images of PHB polymer A. Extracted PHB and B. PHB granules accumulated in the medium. Figure S3. The phase contrast microscope images of biofilm formed on glass slides. A. Control biofilm of V. parahaemolyticus, B. Brevibacterium PHB coated glass slide, C. Standard PHB coated glass slide. [file 12934_2014_114_MOESM1_ESM.docx]

Table S1. Central Composite Design (CCD) design with experimental yield of PHB

| Run A B C D PHB yield (g/L)  1 1.000 1.000 -1.000 -1.000 4.9  2 0.000 0.000 -2.000 0.000 3.9  3 -1.000 -1.000 1.000 1.000 4.5  4 0.000 -2.000 0.000 0.000 4.1  5 2.000 0.000 0.000 0.000 5.7  6 1.000 -1.000 -1.000 -1.000 4.3  7 1.000 1.000 1.000 -1.000 5.1  8 0.000 0.000 0.000 -2.000 3.1  9 0.000 0.000 0.000 0.000 6.74  10 -1.000 1.000 1.000 -1.000 4  11 0.000 0.000 0.000 0.000 6.74  12 0.000 0.000 0.000 2.000 3.9  13 -1.000 -1.000 -1.000 -1.000 4  14 -1.000 -1.000 -1.000 1.000 4.4  15 1.000 1.000 -1.000 1.000 4.5  16 0.000 0.000 0.000 0.000 6.74  17 0.000 0.000 0.000 0.000 6.74  18 -1.000 1.000 -1.000 -1.000 4  19 0.000 2.000 0.000 0.000 4.4  20 1.000 -1.000 -1.000 1.000 4.3  21 0.000 0.000 0.000 0.000 6.74  22 -2.000 0.000 0.000 0.000 4.8  23 1.000 -1.000 1.000 -1.000 4  24 0.000 0.000 0.000 0.000 6.74  25 -1.000 -1.000 1.000 -1.000 3.2  26 0.000 0.000 2.000 0.000 4.3  27 1.000 1.000 1.000 1.000 4.6  28 -1.000 1.000 1.000 1.000 4.7  29 1.000 -1.000 1.000 1.000 4.3  30 -1.000 1.000 -1.000 1.000 3.7 |
| --- |

A: Starch (g/L), B: Incubation time (h), C: Temperature (°C) and D: Salinity (%)

Table S2. ANOVA for response surface quadratic model of PHB yield

| Sum of Mean F p-value  Source Squares df Square Value Prob > F  Model 35.94 14 2.57 94.11 < 0.0001  *A-Starch* *1.17* *1* *1.17* *42.91* *< 0.0001*   *B-Incubation period* *0.40* *1* *0.40* *14.68* *0.0016*   *C-Temperature* *0.050* *1* *0.050* *1.85* *0.1941*   *D-Salinity* *0.40* *1* *0.40* *14.68* *0.0016*   *AB* *0.23* *1* *0.23* *8.27* *0.0115*   *AC* *5.62* *1* *5.62* *0.21* *0.6563*   *AD* *0.46* *1* *0.46* *16.70* *0.0010*   *BC* *0.33* *1* *0.33* *12.12* *0.0033*   *BD* *0.39* *1* *0.39* *14.32* *0.0018*   *CD* *0.28* *1* *0.28* *10.10* *0.0062*   *A2* *3.78* *1* *3.78* *138.74* *< 0.0001*   *B2* *10.59* *1* *10.59* *388.35* *< 0.0001*   *C2* *11.91* *1* *11.91* *436.63* *< 0.0001*   *D2* *17.95* *1* *17.95* *658.03* *< 0.0001*  Residual 0.41 15 0.027  *Lack of Fit* *0.41* *10* *0.041*  *Pure Error* *0.000* *5* *0.000*  Cor Total 36.35 29 |
| --- |

Std. Dev. 0.17 R-Squared 0.9887
Mean 4.77 Adj R-Squared 0.9782
C.V. % 3.46 Pred R-Squared 0.9352

PRESS 2.36 Adeq Precision 29.919

#### Fig. S1. Maximum parsimony phylogenetic tree of MSI04 and their closest NCBI (megaBLAST) relatives based on the 16S rRNA gene sequences. Bootstrap values calculated from 1,000 resamplings using neighbor joining are shown at the respective nodes when the calculated values were 50% or greater.


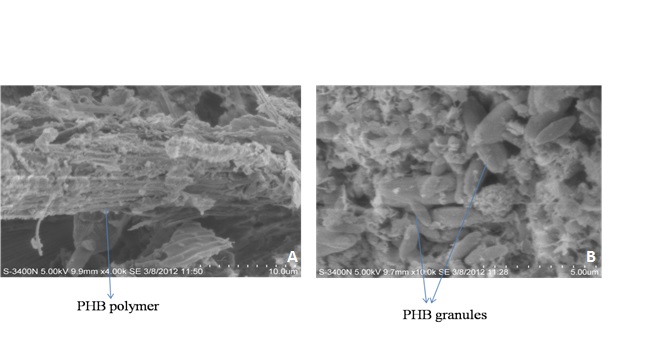


Fig. S2. SEM images of PHB polymer A. Extracted PHB and B. PHB granules accumulated in the medium.


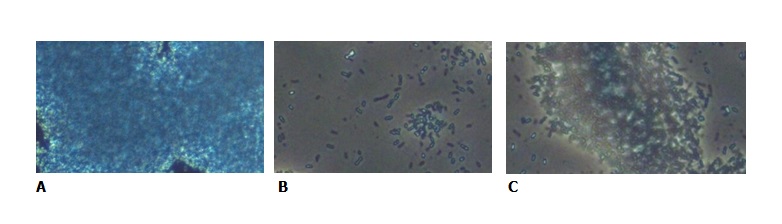


Fig. S3. The phase contrast microscope images of biofilm formed on glass slides. A. Control biofilm of *V. parahaemolyticus*, B. *Brevibacterium* PHB coated glass slide, C. Standard PHB coated glass slide.
